# Mycobacterial SigA and SigB Cotranscribe Essential Housekeeping Genes during Exponential Growth

**DOI:** 10.1128/mBio.00273-19

**Published:** 2019-05-21

**Authors:** Kelley Hurst-Hess, Rajesh Biswas, Yong Yang, Paulami Rudra, Erica Lasek-Nesselquist, Pallavi Ghosh

**Affiliations:** a Division of Genetics, Wadsworth Center, New York State Department of Health, Albany, New York, USA; b School of Public Health, University at Albany, Albany, New York, USA; Washington University in St. Louis School of Medicine

**Keywords:** ChIP-Seq, *Mycobacterium*, rifampin, *sigB*, sigma factor

## Abstract

Mycobacterial σ^B^ belongs to the group II family of sigma factors, which are widely considered to transcribe genes required for stationary-phase survival and the response to stress. Here we explored the mechanism underlying the observed hypersensitivity of Δ*sigB* deletion mutants of Mycobacteriumsmegmatis, M. abscessus, and M. tuberculosis to rifampin (RIF) and uncovered an additional constitutive role of σ^B^ during exponential growth of mycobacteria that complements the function of the primary sigma factor, σ^A^. Using chromatin immunoprecipitation sequencing (ChIP-Seq), we show that during exponential phase, σ^B^ binds to over 200 promoter regions, including those driving expression of essential housekeeping genes, like the rRNA gene. ChIP-Seq of ectopically expressed σ^A^-FLAG demonstrated that at least 61 promoter sites are recognized by both σ^A^ and σ^B^. These results together suggest that RNA polymerase holoenzymes containing either σ^A^ or σ^B^ transcribe housekeeping genes in exponentially growing mycobacteria. The RIF sensitivity of the Δ*sigB* mutant possibly reflects a decrease in the effective housekeeping holoenzyme pool, which results in susceptibility of the mutant to lower doses of RIF. Consistent with this model, overexpression of σ^A^ restores the RIF tolerance of the Δ*sigB* mutant to that of the wild type, concomitantly ruling out a specialized role of σ^B^ in RIF tolerance. Although the properties of mycobacterial σ^B^ parallel those of Escherichiacoli σ^38^ in its ability to transcribe a subset of housekeeping genes, σ^B^ presents a clear departure from the E. coli paradigm, wherein the cellular levels of σ^38^ are tightly controlled during exponential growth, such that the transcription of housekeeping genes is initiated exclusively by a holoenzyme containing σ^70^ (E.σ^70^).

## Abstract (precis)

All mycobacteria encode a group II sigma factor, σ^B^, closely related to the group I principal housekeeping sigma factor, σ^A^. Group II sigma factors are widely believed to play specialized roles in the general stress response and stationary-phase transition in the bacteria that encode them. Contrary to this widely accepted view, we show an additional housekeeping function of σ^B^ that complements the function of σ^A^ in logarithmically growing cells. These findings implicate a novel and dynamic partnership between σ^A^ and σ^B^ in maintaining the expression of housekeeping genes in mycobacteria and can perhaps be extended to other bacterial species that possess multiple group II sigma factors.

## INTRODUCTION

Transcription in bacteria is carried out by a multisubunit RNA polymerase (RNAP) that associates with an interchangeable sigma subunit and directs the transcription machinery to specific promoter regions (1–4). All bacteria encode an essential principal sigma factor and a variable number of alternative sigma factors. Sigma factors are classified into four groups based on the presence of conserved domains 1 to 4. Group I sigma factors are required for transcription of housekeeping genes and are essential (5–7). Group II sigma factors are closely related to those in group I, lack domain 1.1, but are nonessential. Group III sigma factors contain domains 2 to 4, whereas the group IV sigma factors contain only domains 2 and 4. Mycobacteria encode one sigma factor belonging to each of groups I to III and a variable number of group IV sigma factors: 10 in Mycobacteriumtuberculosis, 16 in M. abscessus, and 25 in M. smegmatis (8). Group IV sigma factors have been studied extensively and are involved in heat shock, cold shock, hypoxia, carbon starvation, surface and oxidative stresses, and virulence (8–10). The mycobacterial σ^A^, a group I sigma factor, is essential and highly similar to the primary sigma factors from other bacteria, suggesting that it is the principal sigma factor in mycobacteria (6, 11). σ^A^ mRNA levels are constant under different growth conditions, though the levels of the σ^A^ protein have been seen to decrease during stationary phase (6, 7). The mycobacterial σ^B^, a group II sigma factor, lacks domain 1.1 and shows an ∼64% sequence identity with σ^A^; in fact, residues important for recognition of −10 and −35 promoter elements are identical between mycobacterial σ^A^ and σ^B^ (12, 13). Although it is not essential for survival, σ^B^ is >90% conserved across mycobacterial species. A deletion in *sigB* results in sensitivity to heat, oxidative, and surface stress *in vitro* and an increased sensitivity to *p*-aminosalicylic acid, sulfamethoxazole, and ethambutol but does not impact the survival of M. tuberculosis in macrophages or mouse lungs (10, 12, 14–19). Two attempts to characterize the σ^B^ regulon yielded contradictory results. The global transcription profile of a strain overexpressing σ^B^ identified 72 σ^B^-dependent genes, while the global transcription profile of a Δ*sigB* strain compared to that of wild-type (WT) bacteria identified only 8 σ^B^-dependent genes during exponential growth (12, 14). This disparity can be resolved by determining the binding sites of σ^B^; although a comprehensive map of transcription regulators, including sigma factors, has been determined in M. tuberculosis using chromatin immunoprecipitation sequencing (ChIP-Seq), this does not include SigB binding sites (20, 21). Exposure to diamide and SDS stress resulted in the downregulation of 40 and 72 genes, respectively, in the Δ*sigB* strain compared to their expression in wild-type bacteria (14). Furthermore, the transcription of σ^B^ was shown to occur from two promoters: one recognized by the stress-inducible sigma factors σ^E^, σ^H^, and σ^L^ and the other recognized by σ^F^ (22–25). These observations together led to the general notion that σ^B^ has little role in exponential growth; rather, it is required solely for the mycobacterial response to stress.

RNA polymerase is a target for the broad-spectrum antibiotic rifampin (RIF), which comprises a frontline therapy against M. tuberculosis infection. RIF exerts its effect by binding to the β subunit of RNA polymerase in a region comprising the DNA/RNA channel and sterically blocks the extrusion of elongating RNA when the transcript exceeds 2 to 3 nucleotides (nt) in length (26). High levels of clinically acquired RIF resistance involve *rpoB* mutations in four distinct sequence clusters (clusters N, I, II, and III), the majority of which map to cluster I (27–32). In contrast to acquired resistance, the fast-growing mycobacteria, such as M. smegmatis and M. abscessus, are naturally RIF resistant, albeit to various extents. This intrinsic rifampin resistance has been attributed to the presence of a rifampin ADP-ribosyltransferase (Arr), which inactivates the drug by ribosylation (33–35). The association of RNAP with accessory proteins, such as certain sigma factors and RbpA, has also been shown to influence its susceptibility to RIF. Wegrzyn et al. showed that the Escherichiacoli σ^70^-RNAP is considerably more sensitive than σ^32^-RNAP *in vitro* and *in vivo* and that a deletion of Bacillussubtilis *sigB*, the orthologue of mycobacterial *sigF*, renders the bacteria more sensitive to RIF (43). RbpA, an RNAP binding protein conserved in actinomycetes, has been shown to prevent RIF inhibition *in vitro* (37, 38). While RbpA is essential in mycobacteria, a deletion of RbpA in Streptomyces coelicolor results in RIF sensitivity and a slow-growth phenotype (37, 38). It is unlikely that RbpA is involved in the degradation or efflux of RIF but, rather, modifies RNAP. RbpA interacts exclusively with group I and II sigma factors in *Streptomyces* and mycobacteria and stabilizes the formation of open promoter complexes, thereby enhancing the transcription efficiency of holoenzymes containing σ^A^ and σ^B^. The mechanism by which RbpA confers RIF tolerance is unknown but has been shown to not involve an occlusion of the RIF binding site in RNAP, and its effect is presumably indirect (39, 40).

In the current work, we explore the underlying mechanism of RIF sensitivity of Δ*sigB* mutants of M. smegmatis, M. abscessus, and M. tuberculosis and demonstrate that the RIF sensitivity of Δ*sigB* strains is likely not attributable to the lack of transcription of σ^B^-dependent RIF resistance genes. The study has uncovered that, contrary to previous models, σ^B^ is transcriptionally active during the exponential phase of growth of M. smegmatis and actively transcribes several σ^A^-dependent housekeeping genes. Our results therefore demonstrate an active role for σ^B^ in the exponential phase of mycobacterial growth, in addition to its role as a stress response sigma factor.

## RESULTS

### Deletion of *sigB* results in RIF hypersensitivity in M. smegmatis, M. tuberculosis, and M. abscessus.

**FIG 1:**
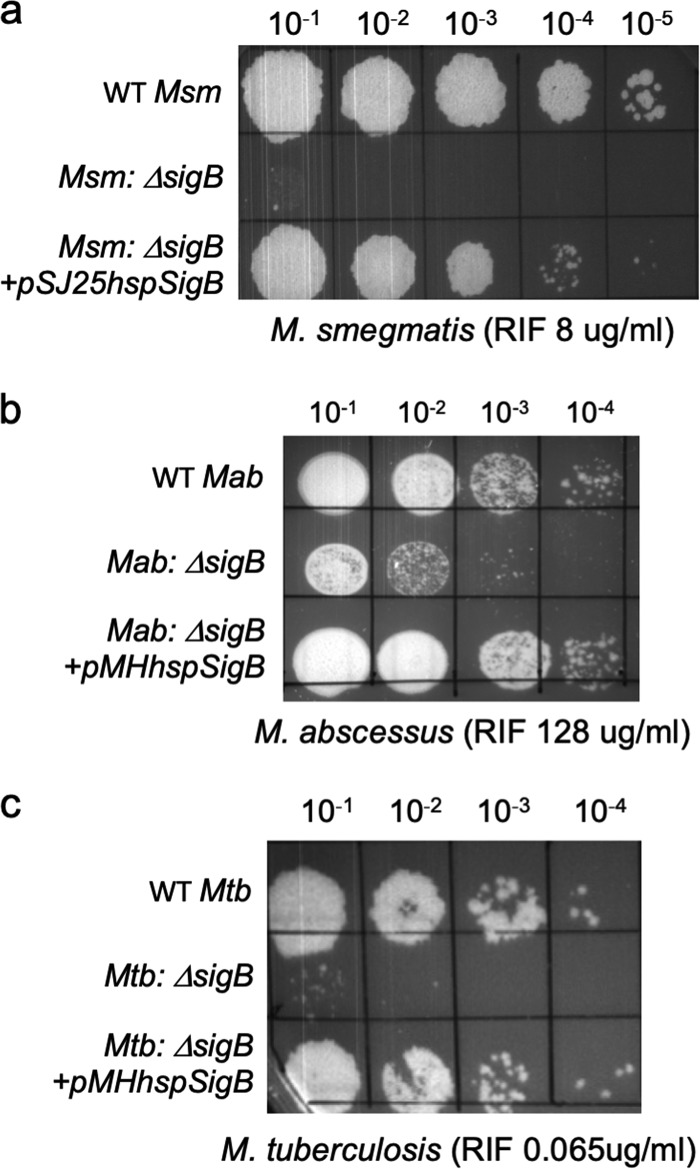
Deletion of σ^B^ confers RIF sensitivity in M. smegmatis (Msm), M. abscessus (Mab), and M. tuberculosis (Mtb). (a to c) Tenfold serial dilutions of M. smegmatis mc^2^155, M. abscessus ATCC 19977, M. tuberculosis mc^2^7000, and their respective Δ*sigB* and complemented strains were grown to an *A*_600_ of 0.7 and spotted on Middlebrook 7H10 ADC or OADC containing the indicated concentrations of RIF. Deletion of *sigB* results in RIF sensitivity in all three strains. The mutant phenotype can be complemented by constitutive expression of the respective *sigB* gene.

To understand the role of sigma factors in mycobacterial drug tolerance, we constructed isogenic deletions in 14 out of 28 randomly selected sigma factor genes in M. smegmatis using recombineering and assayed the sensitivity of the deletion strains to a variety of antibiotics (41). Deletion of the primary-like sigma factor *sigB* resulted in hypersensitivity to RIF (Fig. 1a). We then explored if the phenotype of the Δ*sigB* mutant could be recapitulated in the pathogenic mycobacteria M. tuberculosis and M. abscessus. *sigB* deletion mutations were constructed in the attenuated M. tuberculosis strain mc^2^7000 and the M. abscessus ATCC 19977 strain using recombineering. The Δ*sigB* strains of M. tuberculosis and M. abscessus were found to be hypersensitive to RIF compared to their corresponding wild types (Fig. 1b and c), suggesting that the σ^B^-mediated basal RIF tolerance may be conferred by a conserved mechanism. Growth of the Δ*sigB* mutant of M. smegmatis in Middlebrook 7H10 agar lacking antibiotics was unaffected but was reduced in 7H9 broth compared to that of WT bacteria (see Fig. S1a and b in the supplemental material). The M. tuberculosis Δ*sigB* mutant displayed a slow-growth phenotype on Middlebrook 7H10 agar lacking antibiotics and has previously been shown to exhibit slow growth in liquid media (Fig. S1d) (18).

10.1128/mBio.00273-19.1 FIG S1 Growth of M. smegmatis in media lacking RIF. (a) Tenfold serial dilutions of M. smegmatis mc^2^155, the Δ*sigB* mutant, and complemented strain were grown to an *A*_600_ of 0.7 and spotted on Middlebrook 7H10 ADC or OADC lacking RIF. (b) The growth of M. smegmatis mc^2^155 and the Δ*sigB* mutant in Middlebrook 7H9-ADC-Tween 20 was monitored over a period of 40 h. Deletion of *sigB* results in slow growth in liquid media but no apparent growth change on 7H10 agar. (c) Tenfold serial dilutions of M. abscessus ATCC 19977, M. tuberculosis mc^2^700, and their corresponding Δ*sigB* mutants and complemented strains were grown to an *A*_600_ of 0.7 and spotted on Middlebrook 7H10 OADC medium lacking RIF. Download FIG S1, PDF file, 1.4 MB. Copyright © 2019 Hurst-Hess et al. 2019 Hurst-Hess et al. This content is distributed under the terms of the Creative Commons Attribution 4.0 International license.

### SigB-mediated resistance to RIF is independent of Arr.

**FIG 2:**
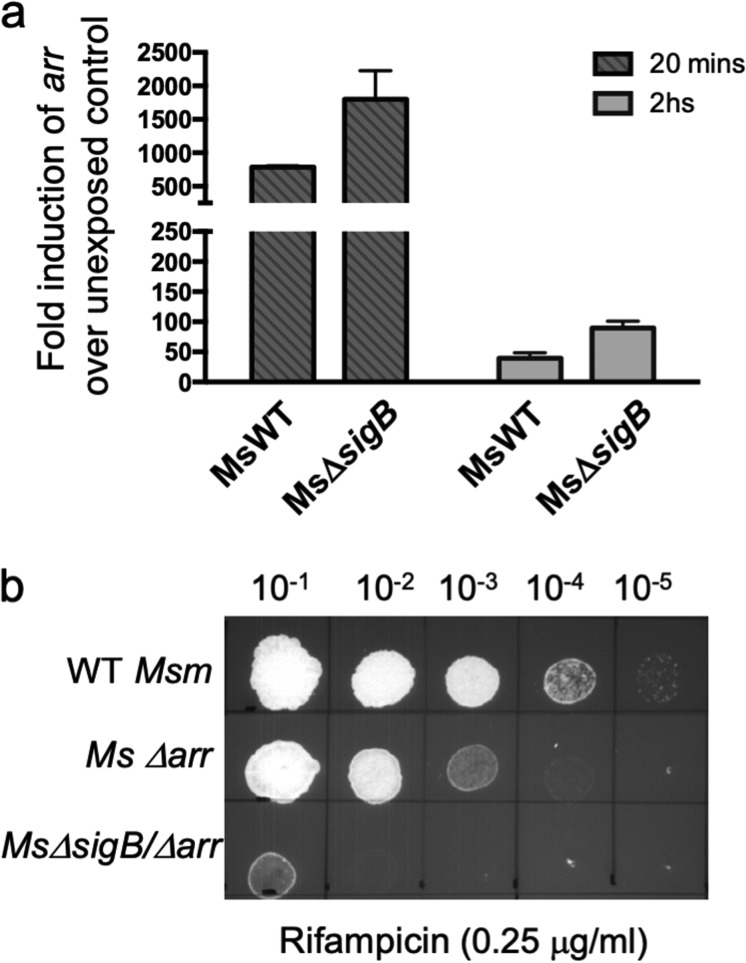
σ^B^-mediated resistance to RIF is independent of ADP-ribosyltransferase (Arr) and putative effector genes. (a) Wild-type M. smegmatis (MsWT) and the *Ms*Δ*sigB* strain were grown to an *A*_600_ of 0.7 and exposed to 4 μg/ml RIF for either 20 min or 2 h, and the amount of M. smegmatis *arr* transcripts was determined by qPCR and plotted as the fold induction over the level of expression for an unexposed control. Data represent the mean ± SD (*n* = 3). *sigA* was used as an endogenous control. (b) Tenfold serial dilutions of WT strain M. smegmatis mc2155 and the *Ms*Δ*arr* and *Ms*Δ*sigB Ms*Δ*arr* strains were grown to an *A*_600_ of 0.7 and spotted on Middlebrook 7H10 ADC containing the indicated concentration of RIF.

Intrinsic tolerance to RIF in mycobacteria has been attributed to the ribosylation of RIF by ADP-ribosyltransferases (Arr), encoded by the fast-growing mycobacteria (42). We first investigated the most likely scenario that σ^B^ is required for the transcription of *arr*, either directly or indirectly, such that a deletion in *sigB* abrogates *arr* expression, resulting in RIF sensitivity. We therefore determined the relative abundance of the *arr* transcript in WT M. smegmatis and the Δ*sigB* mutant of M. smegmatis (the *Ms*Δ*sigB* mutant) upon exposure to RIF by quantitative PCR (qPCR) analysis. Figure 2a shows that the level of *arr* induction upon RIF exposure did not decrease in the Δ*sigB* mutant strain, as would be expected if its transcription were solely dependent on SigB. Instead, *arr* transcript levels increased ∼6-fold in a Δ*sigB* strain and may reflect a compensatory response. Although this does not rule out the possibility that σ^B^ is required for the transcription of *arr*, it suggests the presence of redundant pathways for *arr* expression. Nevertheless, this demonstrates that the RIF sensitivity of the Δ*sigB* strain cannot be attributed to a compromised transcription of *arr.* However, it is possible that the cellular level of the Arr protein is indirectly influenced by the absence of σ^B^. If this were the case, we would anticipate that the RIF sensitivities of the Δ*arr* mutant and the Δ*arr* Δ*sigB* double mutant would be indistinguishable. However, we observed that the RIF sensitivity of the double mutant was significantly higher than that of each of the single mutants, suggesting that their effect is additive and mediated through independent pathways (Fig. 2b; Table 1). Moreover, a Δ*sigB* strain of M. tuberculosis, which naturally lacks *arr*, is also hypersensitive to RIF and provides additional support for the suggestion that the σ^B^-mediated resistance to RIF is independent of ADP-ribosyltransferases.

**TABLE 1:** MIC of RIF for the M. smegmatis WT, Δ*sigB*, Δ*arr*, and Δ*sigB* Δ*arr* strains^*a*^

Strain	MIC of RIF (μg/ml)
WT mc^2^155	10
mc^2^155 Δ*sigB*	2.5
mc^2^155 Δ*arr*	0.25
mc^2^155 Δ*sigB* Δ*arr*	0.0625

a The survival of the M. smegmatis mc^2^155 wild-type, Δ*sigB*, Δ*arr*, and Δ*sigB* Δ*arr* strains was determined in a 2-fold dilution series of RIF in Middlebrook 7H9 medium. The minimum concentration of antibiotic required to inhibit 99% of the growth is shown.

### The RIF sensitivity of the Δ*sigB* mutant is independent of the transcription of known and putative RIF resistance effectors.

**FIG 3:**
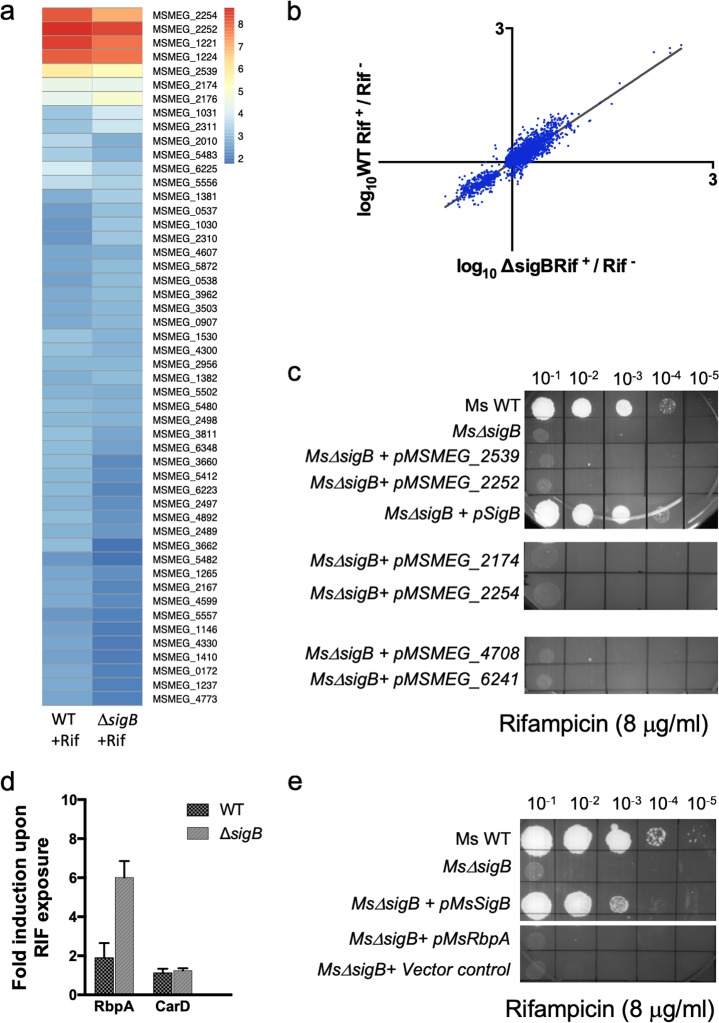
Transcriptomic changes accompanying RIF exposure in the wild-type and Δ*sigB* mutant M. smegmatis strains. (a) Wild-type M. smegmatis and the Δ*sigB* mutant were exposed to 4 μg/ml of RIF for 20 min and analyzed using RNA-seq. Unexposed samples of both strains were used as controls. Two biological replicates of each sample were used. Genes induced >4-fold with a *q* value of <0.001 were analyzed further, and the 50 most induced genes are represented as a heat map. (Left) WT; (right) Δ*sigB* mutant. (b) RIF-induced changes in gene expression in the WT versus Δ*sigB* mutant are shown using genes with a *q* value of <0.1. (c) Complemented strains were created by integrating MSMEG_2539, MSMEG_2252, MSMEG_2254, and MSMEG_2174 (genes highly upregulated in the presence of RIF) and MSMEG_4708 and MSMEG_6241(two SigB-dependent genes identified by RNA-seq) at the Bxb1 *attB* site of mc^2^155 Δ*sigB.* Tenfold serial dilutions of M. smegmatis mc^2^155, the *Ms*Δ*sigB* mutant, and the complemented strains were grown to an *A*_600_ of 0.7 and spotted on Middlebrook 7H10 ADC plates containing the indicated concentration of RIF. Overexpression of the genes listed above did not restore the RIF-sensitive phenotype of mc^2^155 Δ*sigB*. (d) Wild-type M. smegmatis and the *Ms*Δ*sigB* strain were grown to an *A*_600_ of 0.7 and exposed to 4 μg/ml RIF for 30 min, and the amounts of the *rbpA* and *carD* transcripts were determined by qPCR and plotted as the fold induction over the level of expression for an unexposed control. Data represent the mean ± SD (*n* = 3). *sigA* was used as an endogenous control. (e) A strain complemented with *rbpA* was created by integrating MSMEG_3858 at the Bxb1 *attB* site of mc^2^155 Δ*sigB.* Tenfold serial dilutions of M. smegmatis mc^2^155, the *Ms*Δ*sigB* mutant, and the *rbpA* complemented strain were grown to an *A*_600_ of 0.7 and spotted on Middlebrook 7H10 ADC plates containing the indicated concentration of RIF.

We next tested if σ^B^ regulates the expression of genes besides *arr* that mitigate the effect of RIF. We analyzed the transcription profile of wild-type mc^2^155 and the Δ*sigB* mutant upon exposure to sublethal doses of RIF (4 μg/ml) using RNA sequencing (RNA-seq). σ^B^-dependent genes that confer RIF resistance would be detectable as those that are RIF inducible in the wild type but not in the Δ*sigB* mutant. An exposure time of 20 min was found to be most appropriate to enable detection of the gene expression changes that immediately follow RIF exposure. Exposure of wild-type bacteria to RIF caused a >4-fold induction of 101 genes with a *q* value of <0.001 (Data Set S1), of which the top 50 are represented in Fig. 3a, left. The most highly induced genes were MSMEG_2252 (homologue of rifampin monooxygenase [Rox]), MSMEG_2539 (thiopurine methyltransferase), MSMEG_2174 (helicase), MSMEG_2254 (oxalate decarboxylase), MSMEG_1221 (ADP-ribosyltransferases [Arr]), and MSMEG_1224 (Arr). Surprisingly, however, genes that were RIF inducible in wild-type bacteria showed comparable levels of induction in the Δ*sigB* mutant strain (Fig. 3a, right; Fig. 3b; Fig. S2; Data Set S1). Consistent with this observation, the RIF tolerance of the Δ*sigB* strain could not be restored to wild-type levels by overexpression of MSMEG_2252, MSMEG_2254, MSMEG_2539, or MSMEG_2174 (Fig. 3c). Interestingly, although a deletion in MSMEG_2174 increased RIF susceptibility, its expression was unchanged in the Δ*sigB* mutant, indicating that the phenotype is *sigB* independent (Fig. S3).

10.1128/mBio.00273-19.2 FIG S2 Principal-component analysis (PCA) plot generated from RNA-seq data for M. smegmatis mc^2^155 and the Δ*sigB* mutant, unexposed controls, and strains exposed to RIF (4 μg/ml for 20 min) Replicate samples are indicated by the same color. The samples are plotted with respect to their first two principal components, which represent the *x* and *y* axes, respectively, and account for 87% and 7% of the variance, respectively. The PCA plot was generated with the DESeq2 package in R. Download FIG S2, PDF file, 0.5 MB. Copyright © 2019 Hurst-Hess et al. 2019 Hurst-Hess et al. This content is distributed under the terms of the Creative Commons Attribution 4.0 International license.

10.1128/mBio.00273-19.3 FIG S3 Isogenic deletions in MSMEG_2252, MSMEG_2539, and MSMEG_2174 were created by recombineering, and the strains were assayed for RIF sensitivity. The strain with the MSMEG_2174 deletion displayed increased RIF sensitivity compared to WT bacteria; the RIF sensitivities of the strain with MSMEG_2252 and MSMEG_2539 deletions were unchanged. Download FIG S3, PDF file, 1.6 MB. Copyright © 2019 Hurst-Hess et al. 2019 Hurst-Hess et al. This content is distributed under the terms of the Creative Commons Attribution 4.0 International license.

10.1128/mBio.00273-19.7 DATA SET S1 Transcriptome of the M. smegmatis wild type and the Δ*sigB* mutant exposed to 4 μg/ml RIF for 20 min: SD-1 RNA-seq of RIF-induced WT versus SigB.xlsx. Download Data Set S1, XLSX file, 0.02 MB. Copyright © 2019 Hurst-Hess et al. 2019 Hurst-Hess et al. This content is distributed under the terms of the Creative Commons Attribution 4.0 International license.

We also considered the possibility that σ^B^-dependent RIF resistance effectors are constitutively expressed. We therefore compared the transcription profiles of the wild-type and Δ*sigB* strains of M. smegmatis mc^2^155 grown to mid-log phase. RNA-seq analysis showed that 13 genes were significantly (*q* value < 0.01) underexpressed by >3-fold in the mutant (Table S1 and Data Set S2), which is consistent with previously published results (14). We evaluated the role of two of the most highly affected genes that were underexpressed in the Δ*sigB* mutant strain: MSMEG_4708, which encodes a methyltransferase, and MSMEG_6241, which encodes an AAATPase. Overexpression of either of these genes did not complement the RIF sensitivity of the Δ*sigB* mutant strain (Fig. 3c).

10.1128/mBio.00273-19.6 TABLE S1 List of genes downregulated >3-fold in an mc^2^155 Δ*sigB* strain compared to their expression in wild-type bacteria, determined using RNA-seq. Complete data are included in Data Set S2 in the supplemental material. Download Table S1, DOCX file, 0.04 MB. Copyright © 2019 Hurst-Hess et al. 2019 Hurst-Hess et al. This content is distributed under the terms of the Creative Commons Attribution 4.0 International license.

10.1128/mBio.00273-19.8 DATA SET S2 *sigB* regulons of logarithmically grown M. smegmatis: SD-2 RNA-seq of WT versus SigB.xlsx. Download Data Set S2, XLSX file, 0.02 MB. Copyright © 2019 Hurst-Hess et al. 2019 Hurst-Hess et al. This content is distributed under the terms of the Creative Commons Attribution 4.0 International license.

Lastly, we evaluated the role of RbpA, an RNA polymerase-associated protein that has been shown to affect the RIF sensitivity of S. coelicolor and is RIF inducible in mycobacteria (37, 38). Figure 3d shows that RbpA transcript levels increased ∼2-fold in wild-type bacteria and ∼6-fold in the Δ*sigB* mutant upon RIF exposure, consistent with the results of the RNA-seq experiments. Moreover, overexpression of RbpA failed to restore the RIF tolerance of the Δ*sigB* mutant to that of the wild type (Fig. 3e). Together, these observations suggest that the RIF sensitivity of the Δ*sigB* mutant cannot be attributed to the lack of RbpA, a known effector of RIF resistance.

### σ^A^- and σ^B^-containing holoenzymes are indistinguishable in their RIF susceptibility.

Taken together, the data likely rule out the possibility that the RIF-sensitive phenotype of Δ*sigB* is due to the lack of expression of either novel or previously described effectors of RIF resistance. We speculated that the observed RIF sensitivity could be a reflection of the interaction of σ^B^ with RNAP, the target of RIF. The sensitivity of RNAP to RIF has previously been demonstrated to depend on its association with particular species of sigma factors; holoenzymes associated with primary sigma factors are more sensitive than those associated with alternate sigma factors (38, 43). In addition, σ^B^ has been shown to recognize several σ^A^-dependent promoters *in vitro* (13). Based on these two lines of evidence, we propose that the RIF sensitivity of the Δ*sigB* mutant can be explained by one of two scenarios: (i) a holoenzyme containing σ^B^ (E.σ^B^) is more resistant to RIF than a holoenzyme containing σ^A^ (E.σ^A^) and is recruited to housekeeping promoters in the presence of RIF when transcription by E.σ^A^ is compromised, or (ii) E.σ^A^ and E.σ^B^ are equally sensitive to RIF but are both involved in the transcription of housekeeping genes in exponentially growing bacteria. The toxicity of RIF would become pronounced when one of the sigma factors is missing, especially if the expression of neither *sigA* nor *sigB* is inducible. Since σ^A^ is essential, this phenotype is apparent only in a Δ*sigB* mutant.

**FIG 4:**
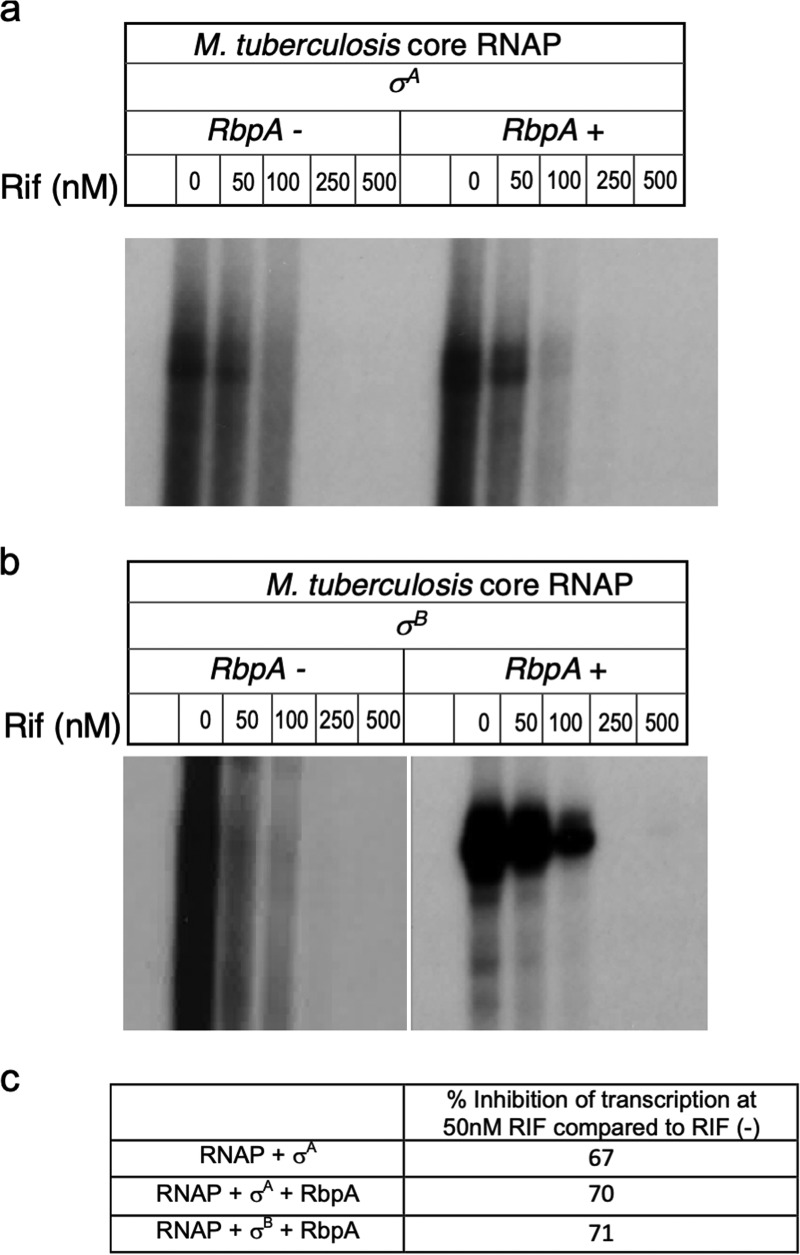
σ^A^ and σ^B^ containing holoenzymes are equally RIF susceptible. (a and b) Multiple-round *in vitro* transcription assays were performed on the *sigA* promoter using 200 nM σ^A^-RNAP/σ^B^-RNAP. RbpA (600 nM) was added where indicated. RIF was added to the indicated concentrations for 30 min at 37°C. Transcription was initiated by addition of 2 μl of an NTP mix (1.5 mM ATP, GTP, and CTP and 0.5 mM UTP) plus 2 μCi of [α-^32^P]UTP. The reaction mixtures were incubated at 37°C for 30 min, and the reactions were terminated by the addition of 5 mM EDTA and 100 μg/ml tRNA. Samples were ethanol precipitated and separated using denaturing PAGE (6% urea polyacrylamide gel). (c) The products were visualized using a Typhoon imager (GE Healthcare) and quantitated using ImageQuant software. Inhibition of RNAP activity at 50 nM RIF is expressed as a ratio of the activity in the presence and absence of RIF.

We determined the RIF sensitivity of σ^A^-RNAP and σ^B^-RNAP by assaying their activity at the *sigA* promoter (*sigAP*) in multiple-round *in vitro* transcription assays. Assays were performed both in the presence and in the absence of RbpA, since RbpA has been shown to assist with open complex formation by σ^A^ and σ^B^, as well as offer protection against RIF inhibition (13, 38–40, 44, 45). Figure 4a and b show that RbpA greatly (>100-fold) enhanced transcription by E.σ^B^, but its effect on E.σ^A^ was modest (∼2-fold) and is consistent with previously published results (13, 40). In the presence of RbpA, although the overall yield of the transcript was ∼100-fold higher when using E.σ^B^, the inhibition of transcription at each RIF concentration was comparable when using either E.σ^A^ or E.σ^B^ (∼70% inhibition was seen with both holoenzymes at 50 nM RIF) (Fig. 4c). This suggests that E.σ^A^ and E.σ^B^ are equally RIF sensitive and that the association of σ^B^ with RNAP does not offer any additional protection against RIF.

### σ^B^ actively transcribes housekeeping genes in exponentially growing M. smegmatis.

**FIG 5:**
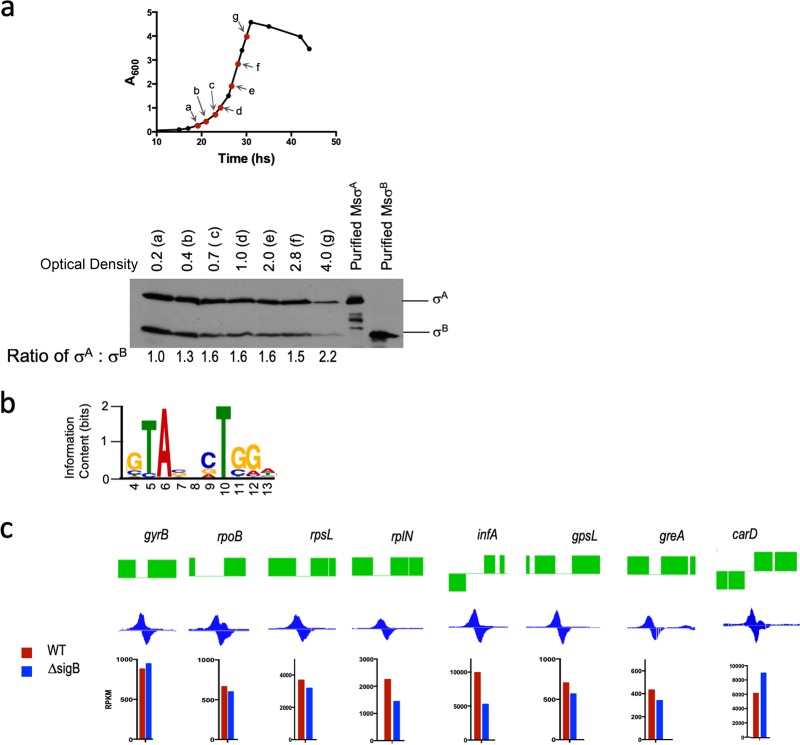
σ^B^ is transcriptionally active in exponentially growing M. smegmatis. (a) (Top) Growth kinetics of wild-type M. smegmatis indicating the growth phase and samples used for Western blotting. (Bottom) Relative levels of the σ^A^ and σ^B^ proteins at the indicated optical densities determined by Western blotting using an anti-σ^70^ monoclonal antibody. Samples were normalized by wet weight and protein concentration to ensure equivalent loading at each OD. Purified σ^A^ and σ^B^ proteins were used as controls. The ratio of σ^A^/σ^B^ was quantitated using ImageJ software and is shown below. Equivalent amounts of protein were loaded in each lane of the Coomassie-stained gel (see Fig. S4b in the supplemental material). (b) Sequence logo of enriched motif in σ^B^-FLAG-bound sites identified using the MEME Suite of tools (MEME E value = 7.0e−003). (c) The ChIP-Seq peaks of σ^B^ bound to promoters of key housekeeping genes visualized with SignalMap software are shown. The transcript levels of the corresponding genes (RPKM values) in the wild type (red) and the Δ*sigB* strain (blue) are plotted.

We next explored the alternate scenario that E.σ^A^ and E.σ^B^ are both involved in the transcription of housekeeping genes in exponentially growing bacteria. This hypothesis is contrary to the currently accepted notion that σ^B^ is required only during transition to stationary phase and in response to environmental stress (7, 8, 12, 14). However, RNA-seq of exponentially growing M. smegmatis showed comparable levels of *sigA* and *sigB* transcripts (Data Set S2) (10). We therefore determined the relative levels of the σ^A^ and σ^B^ proteins at various stages of M. smegmatis growth by Western blot analysis using an anti-σ^70^ antibody that recognizes an epitope in domain 3.1 common to mycobacterial σ^A^ and σ^B^ and E. coli σ^70^ (46) (Fig. S4a). Figure 5a shows that σ^B^ is consistently present in exponentially growing M. smegmatis; in fact, σ^B^ protein levels were comparable to those of σ^A^ during early logarithmic phase of growth.

10.1128/mBio.00273-19.4 FIG S4 (a) Sequence conservation of mycobacterial σ^A^ and σ^B^ and E. coli σ^70^. The conserved epitope recognized by the 2G10 antibody is shown. (b) Coomassie-stained gel serving as a loading control for the assay whose results are presented in Fig. 5a. Equivalent amounts of protein were loaded in each lane. Download FIG S4, PDF file, 1.6 MB. Copyright © 2019 Hurst-Hess et al. 2019 Hurst-Hess et al. This content is distributed under the terms of the Creative Commons Attribution 4.0 International license.

In order to determine if σ^B^ is transcriptionally active in exponentially growing bacteria, we analyzed the genomewide binding profile of σ^B^ in M. smegmatis using chromatin immunoprecipitation sequencing (ChIP-Seq). The *sigB* gene was C-terminally tagged with the 3×-FLAG epitope at its native chromosomal location, grown to mid-exponential phase (optical density at 600 nm [OD_600_] = 0.5), and DNA-nucleoprotein complexes were immunoprecipitated with anti-FLAG monoclonal antibodies. Wild-type strain mc^2^155 lacking a 3×-FLAG fusion was used as a control. Sequenced library reads were mapped to the reference genome using the Bowtie 2 algorithm, and peaks were called using a previously published Python script, Peakcaller (47), and viewed with SignalMap software (Fig. S5a). We identified 327 genomewide peaks of σ^B^ covering 306 genomic regions (peaks within 100 bp of each other were merged), of which 266 peaks were intergenic and 40 mapped within genes (Data Set S3). Transcription start sites (TSSs) for annotated genes in the vicinity of 210 out of the 266 intergenic σ^B^ ChIP-Seq peaks have been previously published; ∼85% of these peaks were located within 11 nt of a TSS and are therefore highly likely to be σ^B^ dependent (Fig. S5b; Data Set S3) (48). σ^B^ binding sites were found to be associated with essential housekeeping genes encoding ribosomal proteins, *rpoB*, *carD*, *gyrA*, and, most prominently, the genes for rRNA. Using the MEME Suite of tools, we could identify a highly enriched motif in 101 σ^B^ ChIP-Seq regions (Fig. 5b) (49). The central core of this motif resembled the −10 consensus sequence 5′-TANNNT-3′ proposed for housekeeping promoters and could be detected in 191 out of 210 intergenic σ^B^ ChIP-Seq peaks found in close proximity to known TSSs (Data Set S3) (8, 50, 51); these included experimentally determined −10 sequences published previously, such as those for *ideR*, *rpsL*, *rrnP*, and *sigA* (52–54). Curiously, however, this motif differed considerably from the −10 consensus 5′-NNGNNG-3′ previously published for M. tuberculosis *sigB*, which could have been a consequence of the methods employed (12). The 5′-NNGNNG-3′ motif was derived using sequence analysis of 5′ untranslated regions of genes that were identified to be σ^B^ dependent using microarrays upon overexpression of σ^B^. The identified genes presumably represent a combination of σ^B^-dependent genes that are transcribed during exponential phase and in response to stress, as well as several additional nonspecific genes that are known to be identified during global transcriptomic analyses using overexpressed proteins (55). The σ^B^ binding sites identified in this study were determined using σ^B^ that was FLAG tagged in its native chromosomal location and were derived from sites that are recognized by σ^B^ only during logarithmic phase. Determination of σ^B^ binding sites under various environmental stresses is likely to identify promoter motifs that differ from the 5′-TANNNT-3′ motif identified here.

10.1128/mBio.00273-19.5 FIG S5 (a) Genomewide binding profile of σ^B^ visualized with SignalMap software. (b) Histogram showing the frequency distribution of distances between SigB ChIP-Seq peak centers and TSSs. Download FIG S5, PDF file, 1.6 MB. Copyright © 2019 Hurst-Hess et al. 2019 Hurst-Hess et al. This content is distributed under the terms of the Creative Commons Attribution 4.0 International license.

10.1128/mBio.00273-19.9 DATA SET S3 ChIP-Seq of σ^B^: SD-3 ChIP-Seq binding sites.xlsx. Download Data Set S3, XLSX file, 0.1 MB. Copyright © 2019 Hurst-Hess et al. 2019 Hurst-Hess et al. This content is distributed under the terms of the Creative Commons Attribution 4.0 International license.

A comparison of the σ^B^ ChIP-Seq data with the RNA-seq data for the Δ*sigB* strain revealed that none of the genes whose promoters were bound by σ^B^ were significantly downregulated in the Δ*sigB* mutant (Fig. 5c; Data Set S3). This supports the idea that promoters that are recognized by σ^B^ during exponential growth must also be recognized by an additional sigma factor, likely σ^A^. A previously published study of σ^A^ binding sites in exponentially growing M. smegmatis was performed using E. coli anti-σ^70^ antibody, which recognizes both mycobacterial σ^A^ and σ^B^; this data set therefore represents a combination of σ^A^ and σ^B^ binding sites. A comparison of the sites bound by σ^70^ with those bound by σ^B^-FLAG showed the presence of at least 541 sites that were bound by σ^A^ alone; these included essential genes, such as those encoding subunits of DNA polymerase III, initiation factor 2 (IF-2), peptide release factor 2, RecO, FtsZ, *rpsS*, etc., and thereby explains the essentiality of σ^A^ (46, 56). The 306 σ^B^ binding sites (identified here using anti-FLAG antibody) comprise a subset of total sites identified by E. coli σ^70^ and likely represent sites that are recognized either by σ^B^ alone or by both σ^A^ and σ^B^. In order to distinguish between these possibilities, we overexpressed a FLAG-tagged σ^A^ using the constitutive *hsp60* promoter from a chromosomally integrated location. However, ChIP-Seq using this strain was highly inefficient. Repeated attempts to add a FLAG tag at the C-terminal end of *sigA* at its native chromosomal location were also unsuccessful, suggesting that the presence of a FLAG tag may compromise the function of σ^A^. Despite the inefficiency of ChIP-Seq using σ^A^-FLAG, we could identify 94 σ^A^ binding sites that were common with σ^B^ binding sites (and that were also recognized by E. coli anti-σ^70^ antibody) and likely represent high-affinity binding sites of σ^A^. Out of the 94 σ^A^ binding sites, 61 were associated with known TSSs, and these therefore constitute the minimum number of promoters that are recognized by both σ^A^ and σ^B^, including promoters for essential genes such as those for rRNA, tRNAs, ribosomal proteins, *sigA*, and *rpoB* (Data Set S3).

### Overexpression of σ^A^ restores the RIF tolerance of *Ms*Δ*sigB* to that of the wild type.

**FIG 6:**
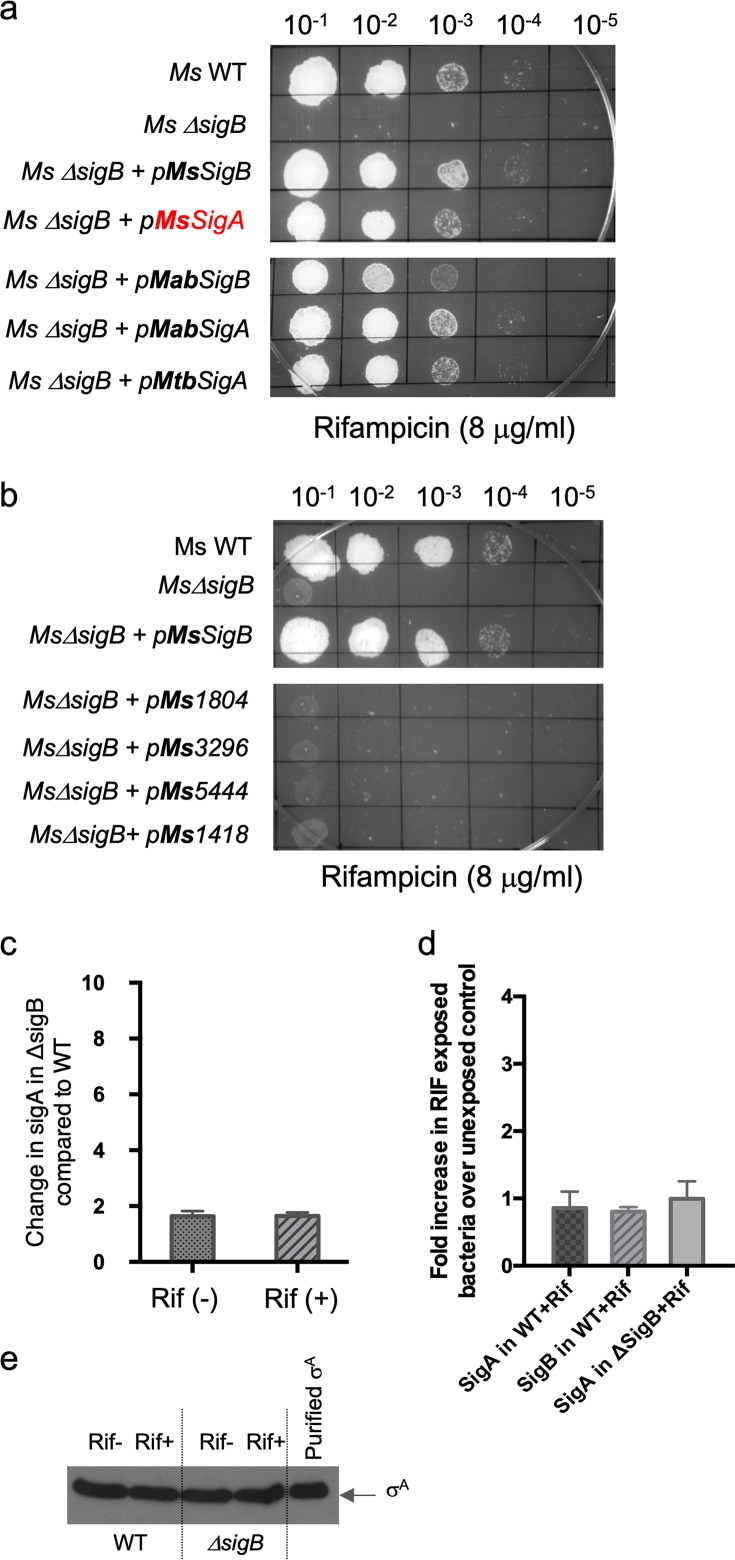
Overexpression of σA restores the RIF sensitivity of the *Ms*Δ*sigB* mutant. (a to c) Complemented strains were created by integrating Ms_SigA, Mtb_SigA, Mab_SigA, Ms_SigB, Mtb_SigB, Mab_SigB, Ms_Ms1804, Ms_Ms3296, Ms_Ms5444, and MSMEG_1418 at the Bxb1 *attB* site of mc^2^155 Δ*sigB*. Tenfold serial dilutions of M. smegmatis mc^2^155, the mc^2^155 Δ*sigB* mutant, and the complemented strains were grown to an *A*_600_ of 0.7 and spotted on Middlebrook 7H10 ADC plates containing the indicated concentrations of RIF. The RIF sensitivity of mc^2^155 Δ*sigB* could be complemented by the constitutive expression of *sigA and sigB* from all mycobacterial strains but not by ECF sigma factors. (b) Wild-type M. smegmatis and the *Ms*Δ*sigB* strain were grown to an *A*_600_ of 0.7 and exposed to 4 μg/ml RIF for 30 min, and the amount of the M. smegmatis *sigA* transcript was determined by qPCR and plotted as the fold induction of *sigA* levels in the *Ms*Δ*sigB* strain over the level of expression in the wild-type strain. The data represent the mean ± SD (*n* = 3). MSMEG_4936 was used as an endogenous control, as its levels were unchanged under various conditions in RNA-seq experiments. (d) Wild-type M. smegmatis and the *Ms*Δ*sigB* strain were grown to an *A*_600_ of 0.7 and exposed to 4 μg/ml RIF for 30 min, and the amounts of the M. smegmatis *sigA* and *sigB* transcripts were determined by qPCR and plotted as the fold induction upon RIF exposure over the level of expression for an unexposed control. The data represent the mean ± SD (*n* = 3). MSMEG_4936 was used as an endogenous control. (e) Wild-type M. smegmatis and the *Ms*Δ*sigB* strain were grown to an *A*_600_ of 0.7 and exposed to 4 μg/ml RIF for 30 min. The levels of σ^A^ protein were determined by Western blotting using an anti-σ^70^ monoclonal antibody. Samples were normalized by wet weight and protein concentration to ensure equivalent loading of each sample. Purified σ^A^ was used as a control.

We reasoned that if transcription of housekeeping genes is initiated by both E.σ^A^ and E.σ^B^, the absence of σ^B^ could be compensated for by increasing the copy number of σ^A^ in a way that mitigates the deleterious effect of RIF. Figure 6a and Table 2 show that the constitutive overexpression of M. smegmatis *sigA* from a chromosomally integrated location restored the RIF sensitivity of the *Ms*Δ*sigB* mutant. Further, we observed that overexpression of either *sigA* or *sigB* from M. abscessus and M. tuberculosis could complement the phenotype of the *Ms*Δ*sigB* mutant (Fig. 6a). However, this effect was restricted to the group I and II sigma factors; *sigF* (group III) and the extracytoplasmic function (ECF) sigma factors were unable to restore the RIF sensitivity of the *Ms*Δ*sigB* mutant (Fig. 6b), presumably because of their inability to initiate transcription at housekeeping promoters. An alternate explanation is that a deletion of *sigB* reduces the levels of σ^A^, which can be complemented by overexpression of σ^A^. We therefore followed the expression of the *sigA* transcript as well as protein levels in the *Ms*Δ*sigB* mutant in the presence and absence of exposure to RIF. Figure 6c and d show that *sigA* transcript and protein levels did not decrease in the *Ms*Δ*sigB* bacteria compared to wild-type bacteria; furthermore, *sigA* expression was also not RIF inducible in either the wild type or the *Ms*Δ*sigB* mutant (Fig. 6e).

**TABLE 2:** MIC of RIF for M. smegmatis wild-type, Δ*sigB*, and Δ*sigB* strains overexpressing either M. smegmatis *sigB* or *sigA*^*a*^

Strain	MIC of RIF (μg/ml)
WT mc^2^155	10
mc^2^155 Δ*sigB*	2.5
mc^2^155 Δ*sigB sigB*_OE_	>10
mc^2^155 Δ*sigB sigA*_OE_	>10

a The survival of M. smegmatis wild-type strain mc^2^155, mc^2^155 Δ*sigB*, and mc^2^155 Δ*sigB* overexpressing M. smegmatis *sigB* and *sigA* (mc^2^155 Δ*sigB sigB*_OE_ and mc^2^155 Δ*sigB sigA*_OE_, respectively) was determined in a 2-fold dilution series of RIF in Middlebrook 7H9 medium. The minimum concentration of antibiotic required to inhibit 99% of the growth is shown.

## DISCUSSION

All mycobacterial species contain a highly conserved group II sigma factor, the product of the *sigB* gene. Global transcriptomic analyses have failed to identify sizable numbers of σ^B^-dependent genes during exponential growth of mycobacteria. Moreover, *sigB* mRNA levels increase upon entry into stationary phase and in response to heat shock and surface and oxidative stresses (5, 7, 10, 14). These observations have led to the inference that σ^B^ is specialized for transcription during transition to stationary phase and in the global stress response but does not play an active role in transcription during the logarithmic phase of growth. Herein we present a series of observations which together illuminate a role for σ^B^ during exponential growth, in addition to it being a stress response sigma factor.

Using ChIP-Seq analysis under exponential growth conditions, we demonstrated that σ^B^ binds to over 200 promoter regions, several of which control the transcription of essential housekeeping genes, such as the rRNA gene, *carD*, *rpoB*, etc. This finding is consistent with that of RNA-seq analysis, which showed that *sigB* mRNA is as abundant as *sigA* mRNA during exponential phase of M. smegmatis growth (see Data Set S2 in the supplemental material) and that the σ^B^ protein is consistently present in all stages of growth (Fig. 5a). A limited ChIP-Seq data set for ectopically overexpressed FLAG-tagged σ^A^ confirmed at least 61 promoter sites that were recognized by both σ^A^ and σ^B^, including those that control the vital cellular functions of ribosome biogenesis and transcription. The most plausible explanation for σ^B^ occupancy at such crucial sites is that it is engaged in active transcription of these genes, or else its occupancy would interfere with the σ^A^-dependent transcription initiation from these sites. These results imply that E.σ^A^ and E.σ^B^ together transcribe a subset of housekeeping genes during exponential growth of M. smegmatis. While further data are required to determine the relative occupancy of E.σ^A^ and E.σ^B^ at a given promoter, we predict that this varies with the promoter site, its association with accessory proteins, as well as with changing growth and environmental conditions. An example of such a scenario has previously been demonstrated *in vitro* by Hu et al., in which E.σ^A^ and E.σ^B^ are transcriptionally active at the *sigAP* but open complex formation on this promoter is seen only with E.σ^B^ in the presence of RbpA, suggesting a higher affinity of E.σ^B^ at the *sigA* promoter (13).

It is noteworthy that the transcript levels of *sigA* as well as σ^A^ protein levels are not responsive to the lack of σ^B^ in a Δ*sigB* mutant strain (Fig. 6c and e); *sigA* is also not RIF inducible in either the wild type or the Δ*sigB* mutant (Fig. 6d). The absence of σ^B^ in a Δ*sigB* mutant strain must therefore result in a decrease in the concentration of holoenzyme that is available for transcription of a subset of housekeeping genes. We would therefore predict a decrease in transcription of some housekeeping genes in the Δ*sigB* mutant strain; however, this was not reflected in our RNA-seq experiments. Since the most prominent binding of σ^B^ is observed at rRNA gene promoters (*rrnP*), it is plausible that the deletion of *sigB* largely impacts transcription from rRNA promoters, changes in which cannot be captured by the RNA-seq approach. Moreover, the rRNA gene promoter has been shown to be particularly susceptible to inhibition by RIF (40). It is therefore tempting to suggest that σ^B^-RNAP plays a crucial role in maintaining the levels of rRNA and that the slow-growth phenotype of Δ*sigB* in liquid media could be a reflection of the decrease in rRNA levels. Addition of RIF to a Δ*sigB* strain could potentially reduce the transcription at *rrnP* further, resulting in growth arrest and the observed sensitivity to RIF. Although we cannot completely rule out the possibility that a σ^B^-dependent gene affects the translation of a RIF effector transcript, we favor an explanation that the addition of RIF targeting a decreased pool of holoenzyme capable of transcribing housekeeping genes contributes to its increased lethality. Restoration of RIF tolerance in the Δ*sigB* mutant by overexpression of σ^A^ further rules out the possibility of a specialized role of σ^B^ in RIF tolerance. The RIF-sensitive phenotype of Δ*sigB* mutants of M. abscessus, M. tuberculosis, and M. smegmatis and the cross-species functional complementarity between σ^A^ and σ^B^ among these species suggest that the role of σ^B^ in the transcription of housekeeping genes during exponential growth is likely to be conserved in all mycobacterial species.

The RNAP-associated protein RbpA has previously been shown to protect against RIF inhibition in S. coelicolor. Although RbpA is incapable of protecting against RIF inhibition at mycobacterial *rrnP* as well as s*igAP*, we and others have noted that the overall levels of transcription by E.σ^A^ at these promoters are higher in the presence of RbpA; moreover, the transcription efficiency of E.σ^B^ at these promoters greatly exceeds that of E.σ^A^ but is strictly RbpA dependent (Fig. 4) (40). Consistent with this observation, a CHIP-Seq analysis of RbpA showed that >75% of σ^B^-bound sites are also bound by RbpA (K. Hurst-Hess, R. Biswas, and P. Ghosh, unpublished results). Curiously, expression of RbpA increases ∼2-fold in wild-type bacteria and ∼6-fold in Δ*sigB* mutant bacteria upon RIF exposure (Fig. 3d). We speculate that in the presence of RIF, the growth of bacteria can be stimulated by RbpA by increasing the transcription efficiency of E.σ^A^ and E.σ^B^ and the increase in RbpA transcription in Δ*sigB* reflects an attempt to compensate for the lack of transcription by E.σ^B^.

The behavior of mycobacterial σ^B^ is reminiscent of that of *rpoS* (σ^38^), a group II sigma factor of E. coli induced during stress and stationary phase with a high degree of similarity to the primary sigma factor of E. coli, σ^70^. σ^70^ and σ^38^ display extensive overlap between their target promoters, and σ^38^ has been shown to take over several housekeeping duties of σ^70^ during stationary phase (57–59). Despite the overlap in promoter recognition, E. coli σ^70^ and σ^38^ have distinct but complementary roles *in vivo*, and σ^38^ transcribes its regulon only under relevant physiological conditions (59, 60). This is achieved in part by tightly regulating the cellular concentration of σ^38^ at the levels of transcription, translation, and proteolysis such that the σ^38^ protein is nearly undetectable during exponential growth but increases during entry into stationary phase (58, 61–63). Additionally, the promoter specificity of E.σ^38^ is modulated to allow transcription of housekeeping genes under appropriate conditions; the precise mechanism by which this is achieved is unclear but may be mediated in part by *cis*-acting promoter features as well as *trans*-acting proteins, such as Crl, an activator that stimulates E.σ^38^ activity at certain promoters, and global regulators like H-NS and IHF (64–68). The association of mycobacterial σ^B^ with RbpA has similarly been shown to allow the recognition of σ^A^-specific promoters by σ^B^ (13). Although the roles of Crl and RbpA appear to be similar, they have been shown to act via distinct mechanisms: Crl increases the affinity of σ^38^ to core RNAP, whereas RbpA stimulates open complex formation without stabilizing the holoenzyme. Mycobacterial σ^B^, nevertheless, presents a clear departure from the E. coli paradigm: the cellular levels of σ^B^ are not controlled during exponential growth, and σ^B^ instead actively participates in the cotranscription of housekeeping genes. Variation in the relative levels of these sigma factors may play a key role in the global regulation of gene expression. We speculate that the presence of σ^B^ may offer an advantage in the survival of mycobacteria under conditions where either the function of σ^A^ is compromised or the bacteria could benefit from the increased transcription of housekeeping genes. Since the expression of σ^A^ itself is noninducible, any demand for increasing the housekeeping gene expression could be achieved by inducing σ^B^ expression. Moreover, we speculate that this mechanism of regulation of gene expression could be more widely utilized, especially in the closely related Streptomyces coelicolor and cyanobacteria that encode multiple group II sigma factors (69).

## MATERIALS AND METHODS

### Media and strains.

Mycobacterium smegmatis was grown at 37°C in Middlebrook 7H9 (Difco) supplemented with 10% albumin-dextrose-catalase (ADC) and 0.05% Tween 20. Mycobacterium abscessus ATCC 19977 was grown at 37°C in Middlebrook 7H9 (Difco) supplemented with 10% oleic acid-albumin-dextrose-catalase (OADC) and 0.05% Tween 20. Mycobacterium tuberculosis mc^2^7000, an attenuated strain of Mycobacterium tuberculosis H37Rv which carries deletions in the RD1 and *panCD* loci, both of which are critical for the virulence of M. tuberculosis, was grown at 37°C in Middlebrook 7H9 (Difco) supplemented with 10% OADC and 0.05% Tween 20 (70). Antibiotics were added as required to the amounts indicated below. Gene replacement mutants were constructed using recombineering as described previously (41). The recombineering construct was generated by cloning in the multiple-cloning sites flanking the apramycin cassette of pYUB854. Mutant clones were checked using the F_check_ and R_check_ primers flanking the deletion site. The *sigB*-FLAG-tagged strain was confirmed using sequencing as well as by Western blot analysis with anti-FLAG antibody.

### Antibiotic sensitivity assays.

Wild-type and mutant strains of M. smegmatis, M. abscessus, and M. tuberculosis were grown to an *A*_600_ of 0.6 to 0.7. Cells were tested for their susceptibility to RIF by spotting a 10-fold serial dilution on Middlebrook 7H10 (Difco) plates containing the concentration of RIF indicated above and below. Antibiotic susceptibility in liquid media was assayed by inoculating the desired strain in a 2-fold dilution series of each antibiotic at an initial *A*_600_ of 0.0004. The cultures were incubated at 37°C, and the *A*_600_ was measured after 48 h for M. smegmatis.

### RNA preparation, qPCR, and RNA-seq analysis.

Wild-type M. smegmatis mc2155 as well as the Δ*sigB* deletion strain were grown to exponential phase (OD_600_ = 0.4) in Middlebrook 7H9-ADC, exposed to 4 μg/ml of RIF for various periods of time (0 to 90 min), and evaluated for the lethality of RIF. Total RNA was prepared from wild-type and mutant strains exposed to RIF (4 μg/ml) for 20 min using a Qiagen RNA preparation kit, followed by DNase I treatment. Unexposed samples were used as controls. Approximately 5-μg total RNA samples were treated by the Ribo-Zero rRNA removal procedure (Illumina) to enrich for mRNA. Approximately 500 ng of RNA was used for library preparation using a ScriptSeq (v2) RNA-seq kit and high-throughput sequencing on an Illumina NextSeq platform. The sequence data were analyzed using the reference-based analysis and default parameters in the Rockhopper (v2.03) program, in which the data are normalized by upper quartile normalization and transcript abundance is reported as the number of reads per kilobase per million (RPKM). Differential gene expression was tested for each transcript, and *q* values, which control the false-discovery rate, were then reported (71, 72). RNA-seq experiments were performed three independent times, using two biological replicates each time.

M. smegmatis wild-type and Δ*sigB* deletion strains were exposed to RIF (4 μg/ml) for the required times. Total RNA was prepared using a Qiagen RNA preparation kit, followed by DNase I treatment. Primers for quantitative reverse transcription-PCR (qRT-PCR) were generated using Primer Quest software (Integrated DNA Technologies). cDNA was generated using random hexamers and Maxima reverse transcriptase (Fisher Scientific), and qRT-PCR was performed using the Maxima SYBR green qPCR master mix (Fisher Scientific) and the following primer pair for MSMEG_1221: 5′-CCTGTGGTTCGCGGAAA-3′/5′-CCCTGCTCAAGAATCTCACC-3′. An Applied Biosystems 7300 real-time PCR system was used with cycling conditions of 50°C for 2 min, 95°C for 10 min, and 40 cycles of 95°C for 15 s and 60°C for 1 min.

### Chromatin immunoprecipitation sequencing and data analysis.

ChIP-Seq was performed as previously described with minor modifications (73). mc^2^155 *sigB*-FLAG was grown at 37°C in Middlebrook 7H9 broth (Becton, Dickinson) supplemented with ADS (albumin [50 g liter^−1^], dextrose [20 g liter^−1^], NaCl [8.1 g liter^−1^]), 0.2% glycerol, and 0.05% Tween 80 to an OD_600_ of 0.4. This was followed by cross-linking with 1% formaldehyde for 30 min with constant agitation and quenching with 250 mM glycine. The cells were pelleted, washed with Tris-buffered saline buffer, and resuspended in buffer 1 containing a protease inhibitor cocktail (73). Cells were lysed on a Covaris S220 Focused ultrasonicator for 30 min (amplitude = 20%, intensity = 5, number of cycles/burst = 200), immunoprecipitated with anti-FLAG monoclonal antibody M2 (Sigma) for 12 h at 4°C, and further processed as described previously (73). Each CHIP-Seq experiment was performed three independent times using two replicates of culture each time.

Genomic DNA libraries enriched for σ^B^ binding were sequenced on the Illumina platform (Wadsworth Center, Sequencing Core Facility). The reads were aligned to the reference genome using the Bowtie2 and SAMtools algorithms (74). Regions of enrichment were identified using a custom Python script as described previously (47). Briefly, for each replicate data set in the pair, an appropriate threshold, *T*_1_ or *T*_2_, was determined for the plus and minus strands. Values for *T*_1_ and *T*_2_ were considered to be between 1 and 1,000. For each combination of values for *T*_1_ and *T*_2_, the number of genome positions with values greater than or equal to the value for *T*_1_ in the first replicate and with values greater than or equal to the value for *T*_2_ in the second replicate was determined. The false-discovery rate was estimated using the null hypothesis that no regions are enriched. The combination of thresholds yielding the highest number of true-positive positions with an estimated false-discovery rate of less than 0.01 was selected. Once *T*_1_ and *T*_2_ were chosen, a region was identified as a peak if both replicates showed enrichment above the corresponding thresholds for each strand. For a peak to be called, there must be a peak on the plus strand within a threshold distance of a peak on the minus strand. Peaks obtained with the Peakcaller program were verified using the MACS2 algorithm and viewed with SignalMap (v2.0.05) software (Roche NimbleGen). Relative enrichment is reported as the fold-over-threshold (FAT) score. The enriched regions were analyzed using MEME Suite (v5.0.3) tools and the default parameters (49).

### Protein overexpression and purification.

M. tuberculosis σ^A^, σ^B^, and RbpA were cloned in pET21a with a C-terminal His tag, transformed into E. coli BL21(DE3)pLysS, grown to an *A*_600_ of 0.4, and induced with 1 mM IPTG (isopropyl-β-d-thiogalactopyranoside) at 30°C. The cells were lysed in a buffer containing 50 mM Tris-HCl (pH 8.0), 300 mM NaCl, and 5% glycerol, and the clarified lysate was loaded on an Ni-nitrilotriacetic acid (NTA) column (Qiagen). Nonspecifically bound proteins were removed by washing with lysis buffer containing 40 mM, 35 mM, and 20 mM imidazole, and the protein was eluted with 150 mM imidazole. For purification of M. tuberculosis RNA polymerase, BL21(DE3)pLysS was cotransformed with pETDuet-Mtbββ′ and pRsfDuet-Mtbαω, grown at 30°C to an *A*_600_ of 0.4, and induced with 0.4 mM IPTG at 16°C for a period of 18 h. The cells were lysed by sonication and passed through an Ni-NTA column (Qiagen) that had been equilibrated with 50 mM Tris, 300 mM NaCl, and 5% glycerol (lysis buffer). The column was washed with lysis buffer and 40 mM imidazole and eluted with lysis buffer and 150 mM imidazole. Fractions containing RNAP were loaded on a heparin-Sepharose matrix (GE Healthcare) that had been equilibrated with 50 mM Tris, 300 mM NaCl, and 5% glycerol and eluted with a buffer containing 1 M NaCl.

### *In vitro* transcription assays.

Multiple-round *in vitro* transcription was performed as previously described (40). In short, 200 nM M. tuberculosis RNAP was assembled with 600 nM the desired sigma factor in a volume of 10 μl for 10 min at 37°C. RbpA (600 nM) was added during assembly to the indicated samples, followed by a further incubation for 5 min. *sigAP* DNA (20 nM) was added to the mixtures, and the mixtures were incubated for 10 min at 37°C. RIF was added to the concentrations indicated below for 30 min at 37°C. Transcription was initiated by addition of 2 μl of a nucleoside triphosphate (NTP) mix (1.5 mM ATP, GTP, and CTP and 0.5 mM UTP) plus 2 μCi of [α-^32^P]UTP. The reaction mixtures were incubated at 37°C for 30 min, and the reactions were terminated by the addition of 5 mM EDTA plus 100 μg/ml tRNA. Samples were ethanol precipitated, resuspended in stop buffer (80% [vol/vol] formamide, 10 mM EDTA, 0.01% xylene cyanol, 0.01% bromophenol blue), and separated using denaturing PAGE (18% urea polyacrylamide gel). The products were visualized using a Typhoon imager (GE Healthcare) and quantitated using ImageQuant software.

### Western blot analysis.

M. smegmatis was grown in Middlebrook 7H9 supplemented with ADS and Tween 20. Aliquots were removed at different stages of growth (*A*_600_ = 0.2, 0.4, 0.7, 1.0, 2.0, 2.8, and 4.0), pelleted, and washed with phosphate-buffered saline (PBS) buffer. Pellets were normalized by weight, resuspended in the required volumes of PBS, and lysed by sonication. The lysate was clarified by centrifugation, the protein concentration was determined at the *A*_260_, and equal quantities of protein from different growth stages were separated using 10% SDS-PAGE, transferred to a polyvinylidene difluoride membrane, and probed with anti-σ^70^ monoclonal antibody 2G10. Purified σ^A^ and σ^B^ were used as controls.
