# Supplementary figures and images for: Mycobacterial SigA and SigB Cotranscribe Essential Housekeeping Genes during Exponential Growth
Source: mBio. 2019 May 21;10(3):e00273-19. doi: 10.1128/mBio.00273-19 (PMC6529629; doi:10.1128/mBio.00273-19)

a

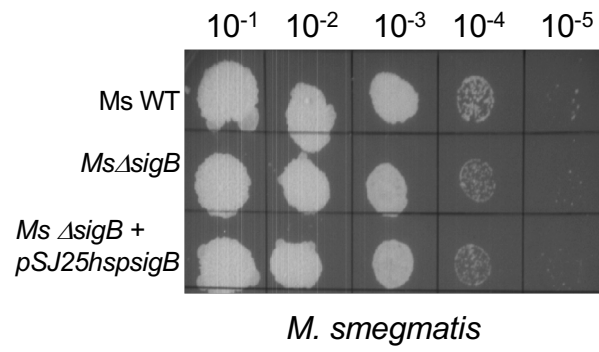

b

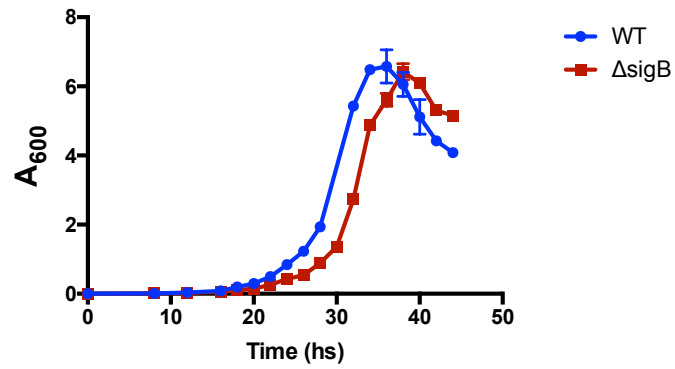

c

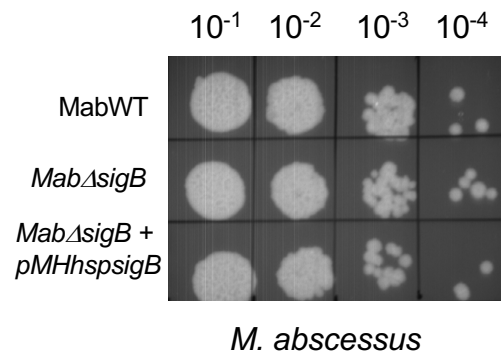

d

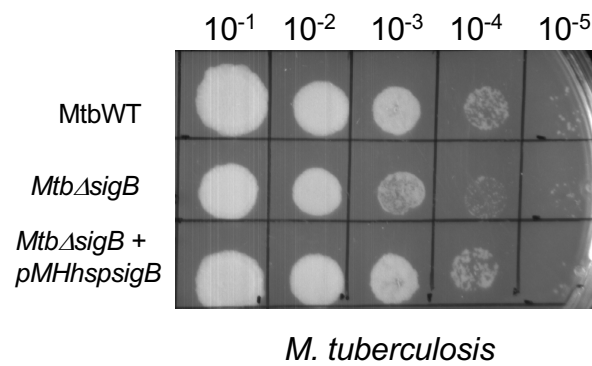

Figure S1

Supplement: FIG S1 [file mBio.00273-19-sf001.pdf]

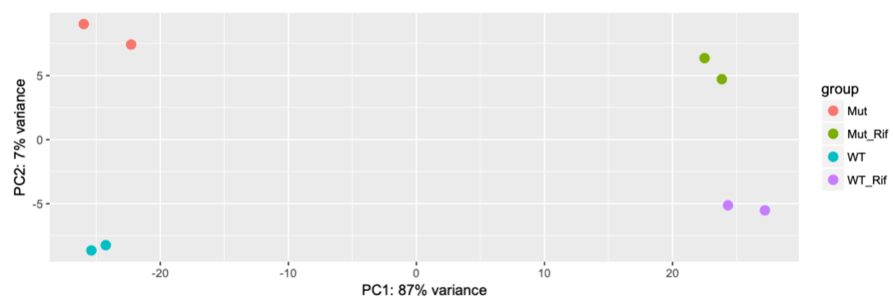

Supplement: FIG S2 [file mBio.00273-19-sf002.pdf]

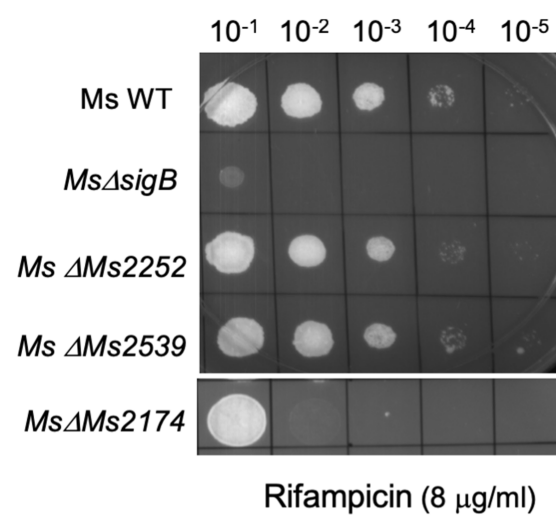

Supplement: FIG S3 [file mBio.00273-19-sf003.pdf]

a

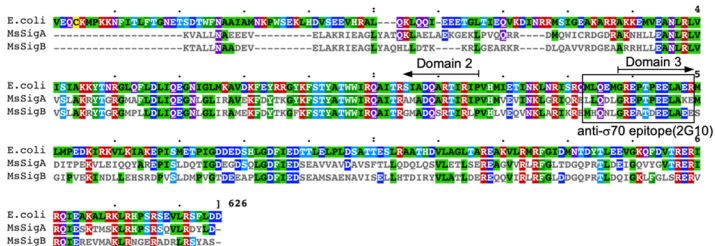

b

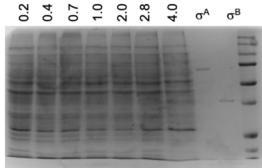

Supplement: FIG S4 [file mBio.00273-19-sf004.pdf]

**a**

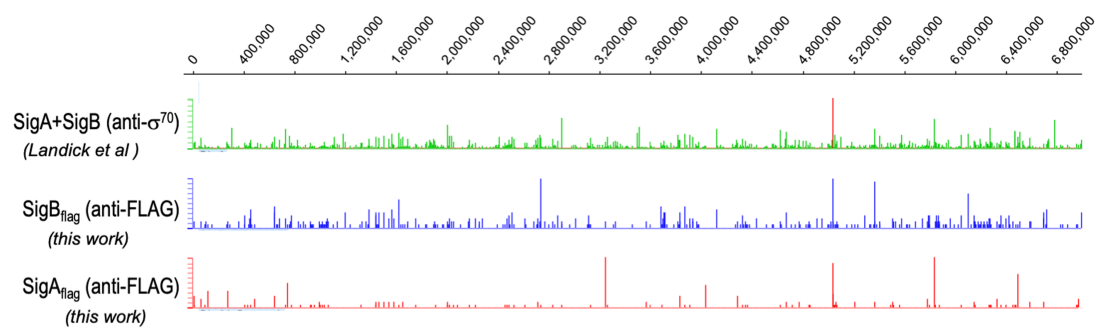

**b**

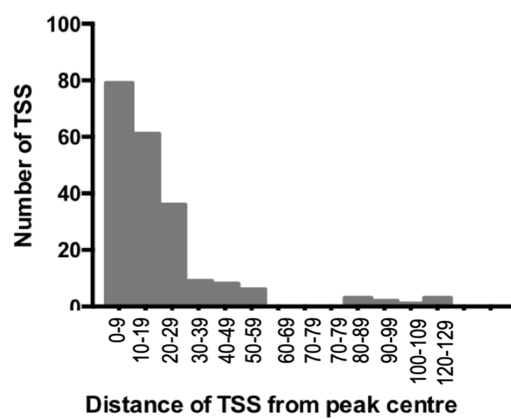

Supplement: FIG S5 [file mBio.00273-19-sf005.pdf]
