# Supplementary material for: Mycobacterial SigA and SigB Cotranscribe Essential Housekeeping Genes during Exponential Growth
Source: mBio. 2019 May 21;10(3):e00273-19. doi: 10.1128/mBio.00273-19 (PMC6529629; doi:10.1128/mBio.00273-19)
Supplement: TABLE S1 [file mBio.00273-19-st001.docx]

**Table S1**: Genes down regulated >3 fold in *M. smegmatis* *ΔsigB*
